# Spousal Kidney Donations in Israel: Religious and Psychoanalytic Perspectives

**DOI:** 10.1007/s10943-025-02497-y

**Published:** 2025-11-19

**Authors:** Moriya Hoter Kariv, Ayelet Oreg

**Affiliations:** The Louis and Gabi Weisfeld School of Social Work, Bar-Ilan University, Ramat Gan, Israel

**Keywords:** Spousal kidney donation, Altruistic donation, Religious motivation, Psychoanalysis

## Abstract

In the existing literature on living kidney donation, most studies address either individual altruistic donations to strangers or familial and friendship-based donations within established social ties. The phenomenon explored here diverges from both: married couples in which each spouse independently donates a kidney to an unknown recipient. This dual altruistic donation, carried out separately yet within the shared framework of marriage, illuminates the intersection of individual moral agency, relational dynamics, and religious faith. Using a qualitative methodology, in-depth interviews were conducted with nine Israeli Jewish religious-nationalist couples (eighteen participants) who each donated a kidney to a stranger. The findings reveal that the decision to donate was motivated by intertwined personal, relational, and faith-based factors. Individually, participants sought meaning, fulfillment, and alignment with moral and spiritual values; relationally, donation reflected partnership, mutual support, and shared ethical purpose; spiritually, it was grounded in Jewish concepts such as *Tikkun Olam* (“world repair”) and *Pikuach Nefesh* (saving a life). A psychoanalytic interpretation drawing on Freud, Klein, and Kohut highlights donation as a process of sublimation, reparation, and self-expansion. The couples’ narratives reveal how altruistic giving can function simultaneously as inner healing, relational repair, and social contribution. The study thus offers an integrated understanding of dual altruistic kidney donation as both a spiritual and relational act of repair, expanding the discourse on altruism, marital intimacy, and faith within contemporary Israeli society.

## Introduction

In recent years, Israel has witnessed the emergence of a unique and expanding phenomenon: married couples in which both spouses have altruistically donated a kidney to a stranger. While such donations can be viewed through the lens of civic engagement, an overlooked but significant form of civic participation (Cnaan & Park, 2015), the present study takes a different approach. We focus on the intersection of religious meaning and psychoanalytic theory, exploring how faith-based values and intrapsychic dynamics shape the lived experiences of married couples in which both spouses have altruistically donated a kidney to a stranger. Although the literature on living kidney donation is extensive and addresses motivations, decision-making processes, and the individual experiences of donors (Lim et al., 2022; Maple et al., 2023; Zuchowski et al., 2023), there remains a significant gap concerning couples who have both undertaken this act. To date, no published studies, in Israel or internationally, have explored the intersection of spousal dynamics, shared decision-making, and dual altruistic donation.

Evidence from *Matnat Chaim* and *Tormim Chaim*, the two principal Israeli nonprofit organizations that promote living kidney donation, as well as findings by Kurleto et al. (2020) and Boas (2019, 2022), indicates that almost all such couples belong to Jewish religious communities, encompassing a range of levels of observance. Within these communities, religious principles, particularly the Jewish concept of *Tikkun Olam* (repairing the world), often frame kidney donation as both a spiritual commandment and a moral duty (Vital et al., 2024).

The present study seeks to examine the experiences of these couples and the meanings they attribute to their joint act of donation, with attention to three interrelated dimensions: the personal experience of each donor, the couple-oriented processes of relational negotiation and shared decision-making, and the influence of religious beliefs, values, and communal contexts.

### Kidney Donation in Global and Israeli Contexts

Over recent decades, living kidney donation has become the most effective treatment for patients with end-stage renal disease, offering significantly higher survival rates than transplants from deceased donors and, in most cases, without compromising long-term donor health (Lim et al., 2022; Nesher et al., 2024). In Israel, altruistic living donation not only addresses the ongoing shortage of transplantable organs but also reflects broader moral, religious, and social values (Boas, 2019; Kurleto et al., 2020). The decision to donate a kidney involves a complex set of ethical, medical, and psychosocial considerations (Marcus et al., 2023). While surgical risks are inherent, many donors report meaningful psychological and spiritual benefits, including enhanced self-worth, deep personal fulfillment, and a strengthened sense of life purpose (Maple et al., 2023; Vital et al., 2024).

Within the broader landscape of kidney transplantation, research distinguishes between directed donations to relatives or acquaintances and nondirected, anonymous donations to strangers (Maple et al., 2023; Zuchowski et al., 2023). The latter form, which is the focus of this study, is associated with distinct emotional, social, and physical complexities. Donors who choose this path often face social misunderstanding, inconsistent professional support, and the challenge of articulating moral reasoning that is highly personal (Maple et al., 2023; Zuchowski et al., 2023). A landmark systematic review by Tong et al. (2012) identified six primary motivations for living kidney donation, ranging from altruism rooted in moral obligation to the pursuit of spiritual validation. The most common of these is a deep concern for the suffering of others, often described as a moral imperative (Clemens et al., 2006). More recent studies have suggested that motivations may evolve over time, becoming integrated into the donor’s personal and relational identity (Pronk et al., 2023).

The process of donation, from the initial decision through recovery, can be demanding both emotionally and logistically. Couples in which only one partner donates have been found to experience changes in relationship dynamics, emotional strain, and even symptoms of depression or anxiety (Clemens et al., 2006; Dasa, 2023). In couples where both spouses donate, these challenges can be compounded by the need for coordinated recovery, mutual caregiving, and adjustments in marital roles. Practical difficulties such as financial strain, workplace disruption, and the need for expanded social support are also common (Dasa, 2023). Moreover, gaps persist in the quality and completeness of information provided to donors compared to recipients, particularly with regard to long-term risks and outcomes (Rota-Musoll et al.,^ 2020^).

Israel is recognized as a global leader in living kidney transplantation. Matnat Chaim, founded in 2009, and Tormim Chaim, established shortly thereafter, have played central roles in raising awareness and facilitating matches between donors and recipients. In 2021, Israel ranked first worldwide in living kidney transplants (IRODAT, 2021). Between 2011 and 2019, the majority of kidney donors in Israel were married, religious Jews, often from smaller or rural communities (Kurleto et al., 2020; Boas, 2022). As of October 2024, twenty-one married couples have each donated a kidney to a stranger (personal communications with the directors of Matnat Chaim and Tormim Chaim). Despite this distinctive and growing phenomenon, there has been no systematic study of these couples’ experiences.

Beyond the Israeli context, regional analyses emphasize the strong influence of religion and social structure on attitudes toward organ donation across the Middle East. Mekkodathil et al. (2020), in a comprehensive meta-analysis, found that although awareness of organ donation is relatively high, willingness to donate remains limited by family norms, religious interpretations, and communal approval processes. These findings situate the Israeli case within a broader cultural framework in which donation is not merely a medical or altruistic decision but a socially negotiated moral act embedded in faith and kinship. Understanding this regional context helps clarify how Israeli donors—particularly those motivated by religious values—reframe organ donation as an act of moral repair and communal responsibility rather than as a purely individual gesture.

### Religious Belief and Practice in the Context of Kidney Donation

Religious frameworks play a central role in shaping the motivations and lived experiences of altruistic kidney donors in Israel. This dynamic has been most clearly illustrated by *Matnat Chaim*, the Orthodox Jewish organization examined by Rabinowich and Jotkowitz (2018). Their study describes how religious leadership and community discourse have transformed living kidney donation into a sanctified act of altruism, framed as both halachically permissible and spiritually desirable. This model demonstrates how faith-based institutions can shift public perceptions of donation from moral hesitation to religious virtue, turning theological conviction into a catalyst for prosocial behavior and communal solidarity.

Boas (2022) found that all Matnat Chaim donors were Jewish, with ninety percent identifying as religious, highlighting the deep influence of faith-based values in guiding decisions about donation. For many donors, the act is not only a personal choice but also a religiously inspired fulfillment of Jewish ethical imperatives such as *Tikkun Olam*, the sanctity of life (*Pikuach Nefesh*), and the mitzvah of saving another (*Hatzalat Nefesh*) (Cooper, 2013; Shapira, 1991; Vital et al., 2024).

Religion operates as both a personal source of meaning and a communal narrative that situates kidney donation within broader ideals of virtue, sacrifice, and service to God (Lazar et al., 2004). In their study of religious behavior in Israel, Lazar and colleagues identified several dimensions of religious motivation: theological motives such as fulfilling divine commandments and seeking closeness to God; ethnocultural motives related to reinforcing Jewish identity; social motives tied to belonging within a community; familial motives that strengthen bonds within the family unit; and traditional motives rooted in the continuation of inherited practices. These dimensions may all converge in the decision to donate a kidney, particularly within religious communities where altruistic giving is celebrated as a moral and spiritual ideal.

Pargament et al. (2000) provide a framework for understanding how religion functions in coping with life stress and decision-making. They outline five interrelated roles: religion as a means of making meaning, offering a sense of control in uncertain circumstances, providing spiritual comfort and a connection to God, fostering closeness to others, and facilitating life transformation. In the context of dual-donor couples, these functions may be experienced not only individually but also as part of a shared marital commitment. The convergence of spiritual devotion and marital partnership can form what might be called a “couple-as-donor” identity—one that embodies the moral and religious virtues of each spouse while reinforcing the unity of the marital bond.

Beyond individual and communal expressions of faith, spiritual leaders play a vital mediating role in shaping donors’ moral and emotional experiences. Carey et al. (2011) describe how healthcare chaplains in Australia act as intermediaries between medical teams, donors, and families, helping to integrate ethical reflection, emotional containment, and spiritual support throughout the donation process. Similarly, in the Israeli context, rabbis often fulfill a comparable pastoral function, guiding donors and their families through the moral complexities of decision-making. Their involvement not only provides theological validation but also fosters psychological resilience, altruistic motivation, and the transformation of donation into a process of spiritual repair and growth.

### Couple Dynamics Through the Lens of Object Relations Theory

Object relations theory provides a framework for understanding how early relationships are internalized and continue to shape adult relationships, particularly in romantic partnerships (Baum, 2006). Freud (2013/1912, 2013/1914) first introduced the concept of transference, whereby feelings, fantasies, defenses, and drives stemming from early childhood relationships, typically with primary caregivers, are often unconsciously projected onto others in the present (Freud, 2013). Klein (2003/1946) expanded on Freud’s ideas with the concept of projective identification, which refers to the process by which parts of the self are either projected onto another or absorbed from the other. These "internalized objects" reflect interpersonal history and are reactivated in significant, intimate relationships such as romantic partnerships (Levita, 2023; Klein, 2003/1937; Bronstein, 2008).

Klein (2003/1937) proposed that in successful romantic relationships, partners often choose a "love object" that mirrors their internalized objects, often coming from similar developmental stages and emotional needs (Klein, 2002). This shared emotional foundation enables both partners to navigate the complexities of love and hate, ultimately internalizing "good objects" that allow for greater attachment, emotional resilience, and the ability to cope with the inevitable disappointments and frustrations inherent in relationships. In relationships characterized by mutual understanding and the ability to express remorse and forgiveness, both partners are able to recognize each other’s uniqueness, which prevents the partner from being reduced to an object of projection. This recognition fosters deeper emotional connection and promotes a healthier, more balanced relationship.

Levita (2023) further expands upon Klein’s ideas by suggesting that romantic relationships can be viewed along a continuum, ranging from relationships characterized by "primitive object relations," which align with the schizoid-paranoid position, to those exhibiting "mature object relations," representing the depressive position. In the schizoid-paranoid position, anxiety about the destruction of the self underpins a defense mechanism of splitting, which is expressed through the dichotomy of "good" versus "bad" (Levita, 2023). This defense mechanism can lead to emotional difficulties in relationships, where intense feelings of frustration, anger, and ambivalence arise, often creating difficulty in forming a coherent, multidimensional experience of the partner. The splitting process also leads to the idealization of the "good" while demonizing the "bad," with the "bad" often experienced as threatening or terrifying (Baum, 2006).

Such primitive object relations hinder intimacy and can result in the projection of emotional content onto the partner. In these cases, one partner may project all their positive traits onto the other while retaining negative emotions within themselves, creating a dependence on the partner as the sole source of goodness and life. This dynamic may limit the autonomy and separateness of both partners, hindering the development of a balanced relationship (Klein, 2003/1946). Furthermore, this dynamic can prevent the recognition of the partner as a separate, independent individual with their own desires, expectations, and needs.

In contrast, the depressive position, as described by Klein (2003/1940), involves a more integrated understanding of the self and the other, where the partner is seen as both good and bad. This understanding allows for the development of greater separateness between the self and the other, fostering independence while maintaining emotional connection. The depressive position also enables a more nuanced and mature emotional experience, where anxiety over losing the partner or their love can be mitigated by the ability to maintain symbolic separation (Klein, 2013/1930).

Mature relationships, then, are characterized by the ability to view the partner as an independent, separate individual, while still maintaining emotional closeness. Partners in these relationships can negotiate and resolve conflicts, navigating a wide range of emotions while balancing intimacy and autonomy (Bronstein, 2008; Klein, 2002). In these relationships, projections are more constructive, facilitating personal integration and promoting greater acceptance of both self and other. The growth of love in mature relationships leads to a more secure sense of self and a deeper connection to the external world, strengthening the capacity to love and allowing both partners to engage in a healthier and more fulfilling partnership (Bronstein, 2008; Klein, 2003/1937; Kohut, 2005).

Object relations theory provides a framework for understanding how early relational patterns shape adult romantic partnerships, influencing how individuals perceive and emotionally invest in one another. Primitive dynamics may lead to dependency and difficulty recognizing the partner’s autonomy, whereas mature dynamics allow for conflict resolution, mutual growth, and balanced interdependence. In the context of dual altruistic kidney donation, this perspective helps illuminate whether the act reflects efforts to repair relational wounds, a shared life project that deepens mutual bonds, or a complex interplay of both. Building on this framework, the present study explores how personal, relational, and religious dimensions intersect in the lived experiences of couples who have each donated a kidney to a stranger.

## Methodology

### Research Design

This study employed a qualitative phenomenological approach to explore the lived experiences of married couples who both chose to donate a kidney to strangers. The phenomenological approach was selected because it facilitates a deep exploration of participants’ subjective experiences and the meanings they assign to these experiences (Creswell, 2013). The approach is rooted in the idea that reality is socially constructed, and individuals’ perceptions of the world are shaped by their personal experiences and the meanings they attribute to them. This approach allows for a comprehensive understanding of how participants interpret and make sense of significant events in their lives, such as donating a kidney to a stranger (Dahal, 2021).

The key principles of phenomenology include the suspension of assumptions and intentionality (Dowling, 2007). The principle of suspension requires the researcher to set aside their preconceptions and biases to fully engage with the participants’ lived experiences, allowing the data to "speak for itself." The principle of intentionality asserts that human experience is always directed toward something specific—every experience is related to thoughts, emotions, and perceptions about particular objects, events, or actions. In this study, intentionality is reflected in the focus on kidney donation, exploring not only the decision to donate but also the personal and social significance of the act, its impact on participants’ relationships, and their retrospective understanding of the experience.

### Participants and Sample

The study included eighteen participants (nine married couples), in which both members chose to donate a kidney to a stranger. Participants were selected using purposive sampling to ensure they represented the phenomenon under investigation. The participants were recruited through (the only) two Israeli organizations, Tormim Chaim and Matnat Chaim, that serve as intermediaries between kidney donors and the National Transplant Center. These organizations provided access to individuals who met the inclusion criteria, which required both spouses to have been married at the time of the donation and at the time of the study, and both spouses to consent to participate in the study and undergo individual interviews. Additionally, snowball sampling (Noy, 2008) was used to further expand the participant pool.

### Research Instruments

The primary data collection method involved semi-structured, in-depth interviews with the participants. Each participant was interviewed separately to ensure that they could express their thoughts and emotions freely without the influence of their spouse, and to allow for a deeper understanding of individual perspectives. The semi-structured interview format was flexible, enabling the researcher to adjust the questions based on the flow of conversation and emerging topics while maintaining a focus on the participants’ lived experiences.

Examples of interview questions included: "What motivated you to consider donating a kidney to a stranger?", "How did you personally experience the kidney donation process?", "What challenges or significant moments did you encounter during the donation process?". The interviews provided insights into the emotional and relational aspects of kidney donation, making it possible to identify patterns, similarities, and differences among participants’ experiences.

### Data Analysis

This study employed a phenomenological thematic analysis to explore the lived experiences of married couples who both donated a kidney to a stranger. The data analysis process followed an iterative approach designed to uncover the essence of participants’ experiences and identify the key themes that emerged from the interviews (Creswell, 2013). Thematic analysis led to identification of patterns and relationships within the data that illuminated the meanings participants attributed to their kidney donation experiences.

The analysis proceeded in two stages. First, we conducted an inductive thematic reading of the transcripts, identifying patterns and recurring motifs grounded in participants’ own words. Meaning units (e.g., significant statements or phrases that captured the essence of participants’ experiences) were extracted from the interview transcripts (Giorgi, 2009). These meaning units reflected the participants’ emotional, relational, and social responses to the donation process and were then grouped into broader themes, including motivations for donation, emotional experiences, relational impacts, and the social and personal significance of donation. These themes were reviewed and refined through multiple iterations to ensure they accurately reflected participants’ lived experiences (Braun & Clarke, 2006).

In the second stage, psychoanalytic concepts were applied to interpret and deepen the thematic analysis, offering insight into unconscious dynamics and relational processes. This sequential process ensured that theoretical frameworks illuminated rather than predetermined the findings.

Throughout the analysis, we employed bracketing, in which the researcher consciously set aside personal preconceptions, assumptions, and biases. According to Moustakas (1994), bracketing is an essential aspect of phenomenological analysis because it allows the researcher to engage fully with the participants’ experiences and minimizes the influence of preconceived notions. Reflexive memos and peer debriefing were used throughout to further enhance analytic rigor and transparency.

### Ethical Considerations

The study followed ethical guidelines to ensure participants’ privacy, confidentiality, and well-being. Participants were informed about the study goals, their voluntary participation, and their right to withdraw at any time. Interviews were conducted via Zoom or in person, based on preference, with a maximum duration of two hours.

To protect privacy, participants were assigned unique identifiers (pseudonyms), and all personal information was kept confidential. Consent forms were signed by participants, detailing the study objectives and their rights. The study received approval from the Ethics Committee of the School of Social Work at Bar-Ilan University (Approval No. 072303).

### Participant Characteristics

All participating couples, except for one in which the husband was religious and the wife secular, identified as members of the national-religious sector. The average age at the time of donation was 41 years. Seven couples resided in peripheral communities, and two couples lived in medium-sized cities. Six of the nine couples in this study resided in the West Bank. While snowball recruitment likely contributed to this concentration, national data confirm that altruistic kidney donors to strangers in Israel are disproportionately concentrated in small, religiously observant communities, many of them located in the West Bank (Nesher et al., 2024). Thus, our sample reflects both recruitment patterns and broader demographic trends.

Educational attainment was generally high: 16 participants held academic degrees and two had earned doctorates. Occupations included education, farming, liberal professions, and public sector positions, and all participants reported active involvement in social initiatives within their communities. In six couples, the husband donated first, while in three couples, the wife donated first. The average time gap between the spouses’ donations was two years, ranging from one month to seven years. Women consistently reported donating after they no longer intended to have children, typically between the ages of 40 and 50. See Table 1 for participant demographics.

**Table 1:** Participant demographic characteristics (N = 18)

Participant	Age at donation	Gender	Religious background	Education	Occupation	PLACE OF RESIDENCE	Donation order	Time gap between spouses’ donations	Additional notes
P1	42	M	National-religious	Academic degree	Educator	Peripheral community (West Bank)	Husband first	2 years	–
P2	39	F	National-religious	Academic degree	Educator	Peripheral community (West Bank)	Husband first	2 years	Donated after finishing childbearing
P3	45	M	National-religious	Academic degree	Farmer	Peripheral community (West Bank)	Husband first	1 year	–
P4	41	F	National-religious	Academic degree	Public sector	Peripheral community (West Bank)	Husband first	1 year	Donated after finishing childbearing
P5	44	M	National-religious	Academic degree	Liberal profession	Medium-sized city	Wife first	3 years	–
P6	43	F	National-religious	Academic degree	Public sector	Medium-sized city	Wife first	3 years	Donated after finishing childbearing
P7	40	M	National-religious	Doctorate	Educator	Peripheral community (West Bank)	Husband first	7 years	–
P8	48	F	National-religious	Academic degree	Public sector	Peripheral community (West Bank)	Husband first	7 years	Donated after finishing childbearing
P9	41	M	National-religious	Academic degree	Farmer	Peripheral community (West Bank)	Wife first	1 month	–
P10	40	F	National-religious	Academic degree	Educator	Peripheral community (West Bank)	Wife first	1 month	Donated after finishing childbearing
P11	39	M	National-religious	Academic degree	Public sector	Peripheral community (West Bank)	Husband first	2 years	–
P12	42	F	National-religious	Academic degree	Public sector	Peripheral community (West Bank)	Husband first	2 years	Donated after finishing childbearing
P13	46	M	National-religious	Academic degree	Educator	Medium-sized city	Husband first	3 years	–
P14	45	F	National-religious	Academic degree	Public sector	Medium-sized city	Husband first	3 years	Donated after finishing childbearing
P15	40	M	Religious husband, secular wife	Academic degree	Liberal profession	Peripheral community (West Bank)	Husband first	2 years	–
P16	41	F	Secular wife, religious husband	Academic degree	Public sector	Peripheral community (West Bank)	Husband first	2 years	Donated after finishing childbearing
P17	44	M	National-religious	Doctorate	Educator	Peripheral community (West Bank)	Husband first	2 years	–
P18	42	F	National-religious	Academic degree	Public sector	Peripheral community (West Bank)	Husband first	2 years	Donated after finishing childbearing

Religious background is based on self-identification. All participants were active in community social initiatives. Ages are rounded to nearest whole year at the time of donation. Women reported donating after they no longer intended to have children (around ages 40–50). Notably, male donors also clustered in the same age range, suggesting that this life stage may be significant for both spouses; this represents an important direction for future research

## Findings

The findings are presented in two overarching themes that reflect the multilayered experience of spousal kidney donation. Theme 1, “Personal and Couple Experience of Donation: Alone and Together,” explores how participants constructed meaning through both individual and relational lenses, emphasizing the interplay between personal motives, spousal dynamics, and shared moral purpose. Theme 2, “The Value and Faith-Based World of the Couple,” examines how the act of donation was embedded in systems of moral values and religious faith, highlighting how participants drew upon spiritual beliefs and communal ethics to guide and sustain their decisions.

While analytically distinct, these two thematic domains are deeply intertwined. Participants’ personal journeys were inseparable from their relationships and from the moral and spiritual frameworks that shaped their worldview. The analysis therefore moves between the personal, relational, and faith-based dimensions to reveal how acts of donation serve simultaneously as expressions of selfhood, marital partnership, and spiritual commitment. Table 2 summarizes the main themes and subthemes identified in the analysis.

**Table 2:** The main themes and subthemes identified in the analysis

Theme	Subtheme	Description and Illustrative Focus
Theme 1: Personal and Couple Experience of Donation—“Alone and Together”	1.1 Alone: Personal Motives in the Decision to Donate a Kidney	Donation as a deeply personal moral act rooted in admiration for other donors, search for meaning, and internal “calling.” Reflects sublimation (Freud) and reparation (Klein)
	1.2 The Act Will Benefit Me: Donation as a Multidimensional Resource	Donation produces personal satisfaction, strengthens couple relationships, enhances family meaning, and expands social belonging. Reflects self-expansion (Kohut) and communal integration
	1.3 Together: The Couple’s Partnership in Donation	Spousal support is essential to decision-making. Couples show either mature dynamics (mutuality and autonomy) or fused dynamics (emotional dependence)
	1.4 Unique Characteristics of the Donors’ Relationships	Marriages marked by mutual respect, autonomy, and friendship. Donation reflects mature relational dynamics aligned with Klein’s depressive position and Kohut’s empathic growth
Theme 2: The Value and Faith-Based World of the Couple	2.1 Doing Good in the World: Donation as an Expression of Values	Donation framed as part of a lifelong moral orientation toward helping others and social contribution. Reflects prosocial values, moral purpose, and community responsibility
	2.2 “And You Shall Find Favor in the Eyes of Your God”: Donation as an Expression of Religious Faith	Religious belief as core motivator and coping resource; donation seen as divine calling and participation in creation. Embodies principles like *pikuach nefesh* and *matan baseter*
	2.3 To Share or Not to Share? The Dilemma of Anonymous Giving vs. Publicizing the Donation	Tension between modesty and advocacy: some prefer anonymity, others share publicly to inspire further donations. Reflects balance between humility and communal responsibility

### Theme 1: Personal and Couple Experience of Donation—“Alone and Together”

This theme explores the multilayered nature of the kidney donation experience, encompassing each participant’s personal journey and the shared dynamics within their marital relationship. It addresses the role of spousal support, mutual encouragement, and the unique form of partnership that develops when both spouses engage in the act of donation.

### Alone: Personal Motives in the Decision to Donate a Kidney

For many participants, the decision to donate a kidney represented a profound personal commitment, emerging from complex internal processes that integrated prior life experiences, moral values, and the aspiration to perform a meaningful act.

Several participants reported that their motivation was sparked by exposure to stories of other donors, which evoked admiration and inspired them to join a community they perceived as embodying exceptional kindness. P1, for example, described experiencing a persistent internal urge over several years: “For about two or three years before the donation, I would hear about people I knew… who donated, and it kind of made me want to… Every time it itched a little, and I thought, maybe one day I’ll do something about it.” (P1)

This sense of belonging to a special group created a deep longing for participation, transforming what once seemed like a distant idea into a tangible, value-aligned action. Similarly, P5 recalled his admiration after encountering media stories about donors:

“I saw various articles… I was unbelievably excited about them [the donors]… I admired them, really… Gradually, I thought, wait, maybe it’s time for me to do this too.” (P5)

For some, seeing others return to full health after donation reduced fears and reinforced the feasibility of the procedure. P10 explained: “You see that [a donor they knew] life goes on as usual, so you say: wow, I have the chance to save someone’s life… and I’ll go back to being who I was.” (P10)

Others framed the decision as part of a personal search for meaning and self-fulfillment. P12 described an immediate sense of identification upon encountering a brochure from *Matnat Chaim*: “I just found it among the newspapers, and the moment I read it, I said: oh, here I am!” (P12)

In these accounts, kidney donation was not only a medical act but also a means of aligning one’s life with deeply held aspirations and values, while joining a wider moral community. These narratives echo Freud’s notion of sublimation, in which inner impulses are transformed into socially valued acts, and Klein’s idea of reparation, whereby unconscious guilt or perceived deficit motivates symbolic repair. The persistent sense of an “itch” or calling described by participants can thus be read as an integration of psychic drive, moral imperative, and communal belonging, situating donation simultaneously as personal healing, ethical fulfillment, and entry into a broader moral world.

### The Act Will Benefit Me: Donation as a Multidimensional Resource

Donation was often described as a multidimensional resource, producing benefits at personal, couple, and family levels. On a personal level, P7 reflected:

“I felt really good about myself, really good and very satisfied that I did something good.” (P7) Similarly, P12 described the presurgery period as a “leap of the heart,” expressing joy and heightened self-worth.

At the couple level, P5 explained that donation “strengthened the relationship” through mutual support, while P8 described it as a “unique shared experience” that allowed quality time for deep conversations, calling it “definitely a gift.”

At the family level, P14 voiced concern over the potential risk of both spouses donating:

“For me, at least, it was clear that we wouldn’t both donate.” (P14).

Her decision was informed by the belief that one spouse should remain available if a child required a kidney in the future. In contrast, P18 viewed donation as enhancing family security: “We know… that every kidney donor (and their family members) actually jumps to the top of the national transplant waiting list.” (P18)

Beyond personal and familial benefits, donation also acted as a bridge between individual and community life. P4 described the overwhelming communal response:

“(After the donation) it was simply a flood of responses from all circles… and a friend’s father… said: ‘Listen, both in what you did and what you said, you’ve lifted the entire community up another level… it was like a tsunami of kindness washing over the community… making everyone want to be just a little bit better at something.’” (P4)

These narratives echo findings by Tong et al. (2012), demonstrating how donation reinforces identity, strengthens social bonds, and generates a collective sense of purpose. Read through the study’s theoretical lenses, this movement from admiration to emulation reflects Klein’s notion of idealization and the internalization of “good objects” (turning admired models into actionable commitments), resonates with Kohut’s account of self-expansion through morally valued action, and aligns with Pargament’s and Lazar’s insights on religion as coping and on social-belonging motives that deepen communal identification and meaning.

### Together: The Couple’s Partnership in Donation

Although donation is ultimately a personal choice, spousal support was consistently described as essential. P5 noted: “At first, I didn’t tell her… but… in order to actually go through with it, I started talking to my wife.” (P5)

P10 emphasized the security provided by her spouse’s encouragement:

“Our relationship… was built together… I knew I had a solid backing… and she was very, very supportive from the beginning.” (P10) Some couples reported that their first donation deepened their bond and motivated a second. P12 reflected:

“The fact that we went through the first donation together… contributed to our willingness to do it again.” (P12) P15 highlighted the strength derived from shared participation:

“When you arrive somewhere as two, it’s an enormous strength; you’re not alone.” (P15)

While these experiences often reflected what object relations theory would term a “mature couple dynamic,” balancing intimacy with autonomy, some accounts could also be interpreted as reflecting a “primitive couple dynamic,” characterized by emotional fusion and projection (Klein, 2002/1957). Taken together, these accounts illustrate how Klein’s theoretical distinction illuminates the varied ways couples navigated the donation process, with some relationships marked by shared strength and mutual support, and others by fusion and dependence.

### Unique Characteristics of the Donors’ Relationships

Participants frequently characterized their marriages as relationships with high autonomy and mutual respect. P7 noted: “If one of us wants to do something, and it doesn’t harm the other, then the other should be happy that they’re doing something good for themselves.” (P7). P18 echoed this sentiment: “We are truly each our own person; each of us has the freedom to do what they want.” (P18). Friendship was also cited as a foundation for mutual support. P12 described: “We have a good friendship… from that place of friendship, we allow differences and choices… we have the desire for the other to achieve their dreams.” (P12). P10 emphasized that their relationship “really allows for very individual lives within the relationship,” underscoring a balance of separateness and togetherness.

Overall, these accounts suggest that for most couples, donation reflected a mature relationship dynamic, in which individuality is preserved while engaging in shared acts of deep meaning (Bronstein, 2008; Levita, 2023). This corresponds to Klein’s description of the depressive position, in which both positive and negative aspects of the partner are integrated, enabling balance between autonomy and intimacy, and supports Kohut’s view of self-expansion, where close relationships provide a foundation for growth through empathic connection and shared purpose.

### Summary of Theme 1: Personal and Couple Experience of Donation

Theme 1 shows that kidney donation was experienced as both a personal commitment and a shared couple project. Individually, participants spoke of an inner calling, admiration for other donors, and the search for meaning, processes that echo Freud’s sublimation, Klein’s reparation and idealization, and Kohut’s self-expansion. Relationally, spousal support was essential, with some couples displaying mature dynamics of autonomy and intimacy, and others showing more fused patterns. Many also described marriages marked by mutual respect and friendship, corresponding to Klein’s depressive position. Together, these findings illustrate how individual motives and couple dynamics intertwined in shaping the act of donation.

### Theme 2: The Value and Faith-Based World of the Couple

This theme explores how kidney donation was embedded in participants’ systems of moral values and religious convictions. For many, the decision was inseparable from a life philosophy centered on altruism, social contribution, and faith in divine providence. The narratives also reveal tensions between the ideal of anonymous giving and the desire—or perceived obligation—to share their stories publicly in order to inspire others.

### Doing Good in the World: Donation as an Expression of Values

Participants frequently framed kidney donation as a direct expression of their commitment to doing good and making a positive impact on society. Many described the act as part of a lifelong orientation toward helping others, often rooted in prior volunteering, social activism, and communal involvement. P12 captured this ethos, describing her donation as:

“A ray of light that reminds people of the goodness in others.” (P12) For her, every good deed had the potential to create “a space of hope,” and donation was both a personal mission and a way of inspiring moral action in others.

P5 expressed a similar sentiment, describing his motivation as a desire “to do something beyond,” positioning the donation as a way to leave a positive mark and improve lives. This desire was not isolated but integrated into his broader moral framework and life goals.

Across accounts, donation was rarely framed as an isolated choice, it was part of a coherent value system emphasizing mutual responsibility, empathy, and proactive contribution to the welfare of others. These findings align with prior research identifying prosocial and value-based motivations in kidney donors (Clemens et al., 2006; Kurleto et al., 2020; Lazar et al., 2004). They also resonate with Pargament’s framework of religion as a source of meaning, control, and transformation, and with Lazar et al.’s typology (2004) of religious motives, where theological, social, and ethnocultural dimensions converge to shape altruistic moral action.

### “And You Shall Find Favor in the Eyes of Your God”: Donation as an Expression of Religious Faith

Religious belief emerged as a central motivator, shaping both the meaning of donation and the emotional resources available to participants during the process.

For some, faith provided a framework for risk acceptance. P5 stated: “If God has decided that we won’t be sick, then even with one kidney, we won’t be sick.” (P5) This reflects a belief in divine providence as a protective force, alleviating anxiety about possible health complications.

P8 expressed a similar conviction, noting that she “trusts in God” and saw His guidance in her decision to donate. For P14, the act was a form of devotion: “All this effort will find favor in the eyes of God.” (P14) Others connected donation to the sanctity of life and the act of creation itself. P6 viewed her donation as a way to “add life,” a particularly meaningful expression for those who had experienced personal loss.

P12 referred to the act as “the miracle of creation,” seeing it as a sacred opportunity to participate in life-giving work. Several participants explicitly linked kidney donation to Jewish religious commandments (*mitzvot*), such as *pikuach nefesh* (saving a life) and *matan baseter* (giving in secret). P15 articulated this as a moral obligation: “What we can fix, we want and need to fix.” (P15)

These testimonies echo findings by Pargament et al. (2000), which highlight the role of religious meaning-making in providing perceived control, strengthening moral commitment, and fostering resilience in the face of uncertainty. They also reflect Lazar et al.’s multidimensional account of religious motives, encompassing theological devotion, ethnocultural identity, and communal belonging, and illustrate how faith-based values provided both an interpretive framework and an emotional resource for participants’ decision to donate.

### To Share or Not to Share? The Dilemma of Anonymous Giving versus Publicizing the Donation

Participants expressed divergent views on whether kidney donation should be a private matter or publicly shared to inspire others. Some, like P15, preferred to keep the act discreet: “There are things I don’t want… to boast about.” (P15)

Others felt a responsibility to share their stories to encourage additional donors. P5 recounted seeking guidance from his rabbi, who advised that publicizing the donation would serve the greater good. Although initially uncomfortable with personal exposure, he accepted the advice and shared his experience. P18 actively embraced the role of an advocate, explaining that sharing her story could “bring more and more donors.” P17 described promoting donation among colleagues, calling it “something very meaningful.”

In some cases, advocacy extended to media projects. P14 and P15, for example, documented their journey in a film to raise awareness and encourage others to consider donation. They saw themselves as “ambassadors” or even “missionaries” for the cause, intentionally using personal narratives to expand the donor community. Taken together, these accounts highlight a continuum between discretion and public advocacy, ranging from those who preferred to keep donation private to those who actively embraced the role of public messengers seeking to expand the circle of donors.

### Summary of Theme 2

Theme 2 highlights how kidney donation was framed as a moral and spiritual act rooted in systems of kindness, responsibility, and faith. Religious belief provided both motivation and coping, consistent with Pargament’s view of religion as meaning-making and resilience, and with Lazar’s account of multidimensional religious motives. While participants did not always articulate their actions explicitly in terms of Tikkun Olam, their narratives reflected its underlying ethos, emphasizing repair, responsibility, and the sanctity of giving. In this sense, Tikkun Olam was implicitly present in participants’ meaning-making, even if not always named directly. Participants also navigated the tension between anonymity and advocacy, reflecting values of humility alongside a sense of communal responsibility to inspire others.

## Discussion

Building on the psychoanalytic understanding of couples and relational dynamics as conceptualized by Klein (2003), the present study explored the experiences of married couples who donated a kidney to a stranger, examining the significance of donation on personal, relational, and faith-based levels. Previous research on individual donors (Clemens et al., 2006; Tong et al., 2012) has shown consistent patterns of personal motivations and experiences. In our study, the personal accounts of spousal donors echoed these findings, suggesting similarities at the individual level, even as relational dynamics introduced new dimensions.

### Relational and Motivational Patterns in Couples’ Decisions

Interviews with participants revealed relationships composed of two strong, decisive individuals whose choices were driven by an internalized faith in personal values. The initial decision of one partner to donate resonated within the family unit and prompted the other partner to follow. Mutual commitment to the values of giving and donation, along with a desire to belong to the donor community and encounters with the stories of nearby donors, served as central motivators for joining the circle of donation. The religious dimension, both in the individual’s actions and in the couple’s shared actions, emerged as a central influence in decision-making. The findings indicate that, although personal and relational aspects were significant, the faith-based dimension was the most salient for participants. Thus, while the couple’s relationship, as understood through Klein’s theories and object relations frameworks, shaped the decision-making process, the religious, ethical, and spiritual experience provided the core motivation behind the act of donation.

Beyond our sample, national patterns also highlight the centrality of religious identity in altruistic kidney donation to strangers. A recent nationwide study of 749 altruistic donors to strangers found that 88% identified as religious or ultra-Orthodox, compared with only 21% of the general population (Nesher et al., 2024). Yet within this highly religious donor population, national-religious participants are disproportionately represented, whereas ultra-Orthodox participation remains limited. This contrast may be explained by differences in community ethos: National-religious populations are marked by strong communal networks, high military service rates, and ideological commitments to sacrifice and civic responsibility, while ultra-Orthodox communities often prioritize insularity and religious study over civic engagement.

### Faith and Moral Imperatives in Altruistic Giving

Although participants did not always use the explicit language of Tikkun Olam, their narratives reflected its central ethos. Accounts of “repairing what can be fixed” (P15), “adding life” (P6), and inspiring others through “a ray of light” (P12) illustrate how participants understood their donation as both personal healing and social contribution. These expressions resonate with Jewish ethical imperatives such as pikuach nefesh (פיקוח נפש saving a life) and matan baseter (מתן בסתר giving in secret), which were explicitly mentioned by some participants. Taken together, these findings suggest that, even when not named directly, the underlying logic of Tikkun Olam informed the way participants framed their decisions and integrated them into systems of faith and responsibility.

This observation is consistent with previous research showing that altruistic kidney donors in Israel often situate their actions within broader religious and communal frameworks (Boas, 2019, 2022; Kurleto et al., 2020). It also aligns with Pargament et al.’s (2000) account of religion as a source of meaning and resilience, and with Lazar et al.’s (2004) typology of religious motives that intertwine theological, social, and ethnocultural dimensions. The current study extends these insights by showing how couples integrate such values into shared life projects, where the donation becomes not only a medical act but a spiritual and communal endeavor.

By situating participants’ testimonies within this broader interpretive frame, their actions can be understood as implicitly grounded in Tikkun Olam: a moral and spiritual commitment to repair, responsibility, and contribution to others. This grounding in participant data provides the foundation for the psychoanalytic exploration that follows.

### A Psychoanalytic Understanding of Tikkun Olam

The concept of Tikkun Olam can be understood psychoanalytically by integrating key theories: Freud’s (2007/1915) sublimation, Klein’s (2003) reparation, Kohut’s (2005/1984) self-expansion, and Netzer’s (2014) spiritual emulation of the divine. Together, these perspectives illuminate kidney donation as both internal repair and social contribution.

#### Freud’s Perspective: Sublimation as Transformative Repair

Freud’s psychoanalytic framework explains how internal drives can be redirected into socially valued actions through sublimation. In the context of kidney donation, sublimation channels potent often unconscious desires into an act that is personally fulfilling and socially commendable. Internal conflicts and repressed impulses are transformed into an expression of compassion and social responsibility, producing a dual repair—mending internal psychic fissures while alleviating another’s suffering. This transformation exemplifies Tikkun Olam as both personal healing and societal contribution.

#### Klein’s Contribution: Reparation and the Dynamics of Object Relations

Klein’s theories focus on early relational experiences and the intrinsic need for reparation. Infants internalize ambivalent feelings toward primary caregivers, producing guilt and a drive to repair perceived damage. In adulthood, kidney donation may serve as a symbolic act of reparation for earlier internal conflicts or relational ruptures. Giving the “gift of life” becomes a way to restore a sense of wholeness, fulfilling both emotional needs and altruistic intentions.

#### Kohut’s Self-Psychology: Expansion, Empathy, and the Transcendent Self

Kohut’s theory emphasizes the evolution of the self through empathy and self-expansion. For kidney donors, the act of giving extends beyond personal gratification, enabling a deeper sense of interconnectedness and affirming self-worth in relation to the collective good. This expansion fosters self-healing and self-realization, where personal repair and social contribution are inseparable.

#### Netzer’s Perspective: Emulating the Divine and the Spiritual Dimension of Tikkun Olam

Netzer (2014), influenced by Jungian thought, adds a spiritual dimension, emphasizing the aspiration to emulate divine qualities. In Jewish tradition, Tikkun Olam entails embodying mercy, compassion, and justice (Kahana, 2020). Kidney donation becomes a tangible expression of spiritual ideals, allowing donors to mirror divine benevolence and elevate their moral standing. Through this act, they address a tangible human need while pursuing spiritual refinement.

### Integrative Reflections and Implications

Taken together, these psychoanalytic perspectives present a multilayered understanding of kidney donation as Tikkun Olam. Internal drives (Freud) are sublimated into altruistic acts; the need for reparation (Klein) is fulfilled through the symbolic gift of life; empathy and self-expansion (Kohut) enable transformative connection; and the aspiration to embody divine qualities (Netzer) infuses the act with spiritual depth. The couples’ joint decision to donate represents both an intimate relational commitment and a manifestation of a broader ethical and spiritual vision. Their journey reflects movement from internal conflict toward external wholeness, illustrating that personal healing and societal betterment are deeply interconnected.

The psychoanalytic contribution at the intersection of religion and health can be further illuminated through Rizzuto’s (1974) object relations approach to the formation of God representations. Rizzuto conceptualizes the image of God as an internalized parental object that evolves from early experiences of love, dependency, and authority. From this perspective, the religious meanings attached to altruistic acts, such as spousal kidney donation, can be understood as reparative processes directed toward these internalized figures. Within this framework, religiously motivated altruism functions not merely as a defensive mechanism or a moral duty, but as a potential source of resilience, repair, and integration. By transforming dependency and guilt into creative acts of care, donors engage in a process that intertwines spiritual growth with emotional healing.

### Limitations

This study has several limitations that should be acknowledged. First, the research question—exploring participants’ experiences on personal, relational, and faith-based levels—may have encouraged participants to present an overly positive self-image, potentially masking internal conflicts, relational tensions, or struggles of faith. To address this limitation, the researchers used indirect and open-ended questions to invite more nuanced and emotionally authentic responses. Follow-up questions were employed to elicit deeper reflections and encourage discussion of challenges as well as strengths.

Second, a cultural and religious gap existed between the secular researchers and the religious-national participants. This difference may have influenced the level of openness or the framing of moral and spiritual concepts. To minimize this limitation, the research team engaged in regular reflexive discussions and maintained reflective journals after each interview to separate personal assumptions from participants’ perspectives. Team debriefings were also conducted to ensure interpretive consistency and sensitivity to participants’ cultural frameworks.

Finally, the interviews were conducted at a single point in time; thus, long-term changes in participants’ relationships or perceptions of donation could not be examined. Future longitudinal research may deepen understanding of how the meanings of altruistic donation evolve over time.

Despite these limitations, the study offers important insights into the intersections of altruism, faith, and relational dynamics in spousal kidney donation, forming the basis for the conclusions that follow.

## Conclusion

Through the lenses of Freud, Klein, Kohut, and Netzer, kidney donation by couples emerges as a complex, integrative process of repair. It addresses psychological needs through sublimation, reparation, and self-expansion, while serving as a concrete expression of the ethical and spiritual ideal of Tikkun Olam. This process intertwines personal transformation with social responsibility, showing that self-change and world-change can occur in tandem. The findings enrich our understanding of altruism, couple dynamics, and faith, revealing giving as a profound act of world repair that blends inner psychological growth with tangible societal benefits.
